# Microenvironmental stimuli induce different macrophage polarizations in experimental models of emphysema

**DOI:** 10.1242/bio.040808

**Published:** 2019-04-10

**Authors:** Júlia Benini Kohler, Daniela Aparecida de Brito Cervilha, Alyne Riani Moreira, Fernanda Roncon Santana, Talita M. Farias, Maria Isabel Cardoso Alonso Vale, Milton de Arruda Martins, Carla Máximo Prado, Iolanda Calvo Tibério, Juliana Tiyaki Ito, Fernanda Degobbi Tenorio Quirino dos Santos Lopes

**Affiliations:** 1 Department of Clinical Medicine, Laboratory of Experimental Therapeutics (LIM-20), School of Medicine, University of São Paulo, São Paulo 01246-903, Brazil; 2 Department of Biological Science, Federal University of São Paulo, Santos 09972-270, Brazil; 3 Department of Bioscience, Federal University of São Paulo, Diadema 09961-400, Brazil

**Keywords:** Macrophages polarization, M1-like phenotypes, M2-like phenotypes, Pulmonary emphysema, Animal model

## Abstract

Macrophages play a pivotal role in the development of emphysema and depending on the microenvironment stimuli can be polarized into M1- or M2-like macrophage phenotypes. We compared macrophage polarizations in cigarette smoke (CS)- and porcine pancreatic elastase (PPE)-induced emphysema models. C57BL/6 mice were subdivided into four experimental groups. In the PPE group, animals received an intranasal instillation of PPE (0.677 IU); in the saline group, animals received an intranasal instillation of saline (0.9%). Animals from both groups were euthanized on day 28. In the CS group, animals were exposed to CS for 30 min, twice a day, 5 days per week for 12 weeks. In the control group, animals received filtered air. We observed an increase in total macrophages for both experimental models. For M1-like macrophage markers, we observed an increase in TNF-α^+^ and IFN-γ^+^ cells, *Cxcl-9* and *Cxcl-10* expressions in PPE and CS groups. Only in the CS group, we detected an increased expression of *IL-12b*. For M2-like macrophages markers we observed a down regulation in IL-10, IL-4, IL-13, *Arg1* and *Fizz1* and an increase of TGF-β^+^ cells in the PPE group, while for the CS group there was an increase in TGF-β^+^ cells and IL-10 expression. All exposure groups were compared to their respective controls. In summary, we demonstrated that CS- and PPE-induced models resulted in different microenvironmental stimuli. CS exposure induced an environmental stimulus related to M1- and M2-like macrophage phenotypes similar to previous results described in COPD patients, whereas the elastase-induced model provided an environmental stimulus related only to the M1 phenotype.

## Abstract (precis)

**Summary:** The CS-induced model showed M1 and M2-related genes and chemokines, whereas the PPE-induced model showed a downregulation of M2- and an increase in M1-related genes and chemokines.

## INTRODUCTION

Chronic obstructive pulmonary disease (COPD) is an inflammatory disease characterized by an exacerbated inflammatory response and/or alveolar abnormalities usually caused by constant exposure to noxious particles and gases, most commonly due to cigarette smoke (CS) (GOLD, 2019; Hodge et al., 2007).

Macrophages play a pivotal role in the clearance of exogenous particles, in the defense against pathogens of the respiratory tract and are recognized as important mediators of the inflammatory response in COPD. An increased number of macrophages in lung and sputum samples have been found in COPD patients (Grashoff et al., 1997) and the degree of the disease has been shown to be associated with the extent of the increase in these cells (Finkelstein et al., 1995; Hogg et al., 2004).

Depending on the microenvironment stimuli, macrophages can be polarized into M1- or M2-like phenotypes with different functional capacities (Shaykhiev et al., 2009). M1 polarization occurs in the presence of interferon (IFN)-γ or by exposure to pathogen components (Goerdt and Orfanos, 1999; Mantovani et al., 2004) during the Th1-mediated immune response, exerting cytotoxic and antitumoral functions.

In contrast, M2 polarization is induced by exposure to Th2 cytokines such as interleukin (IL)-4 and IL-13 (M2a polarization) or by combined exposure to immune complexes and toll-like receptor (TLR) or IL-1R agonists (M2b polarization), showing immunoregulatory functions. In the presence of IL-10 (M2c polarization) macrophages are able to suppress immune responses and stimulate tissue remodeling (Mantovani et al., 2004).

Animal models are widely used to provide better mechanistic insights into the pathogenesis of COPD (Vlahos et al., 2006) however, it is important to understand the advantages and disadvantages of each model.

The CS and elastase-induced models are the most commonly used models since they are physiologically similar to the human disease. However, there are important differences between both models in terms of the inflammatory profile, the level of alveolar destruction and the required time to disease development (Wright et al., 2008).

Exposure to CS is considered the best experimental model at mimicking the pathogenesis of the human disease, since smoking is still considered the main cause of the development of COPD. However, a disadvantage of this model is that although it requires a long time of exposure, the resulting alveolar enlargement is mild in comparison with that resulting from animal models of protease instillation (Wright et al., 2008; Lopes et al., 2013; Mahadeva and Shapiro, 2002).

In contrast, the elastase-induced model, depending on the doses, induces quicker and greater alveolar destruction than that in the CS model (Bonfield, 2012). However, a major disadvantage of this model is that it does not trigger all the physiological events observed in the CS models (Wright et al., 2008).

Considering the importance of macrophages in the development of COPD (Barnes, 2004) and bearing in mind that there are differences in the pathophysiological mechanisms between both mostly used experimental models, we aimed, in this study, to evaluate how the differences in microenvironment stimuli could interfere with macrophage polarization, by comparing the elastase and the CS-induced models.

## RESULTS

### Lung morphometry

**Fig. 1.**
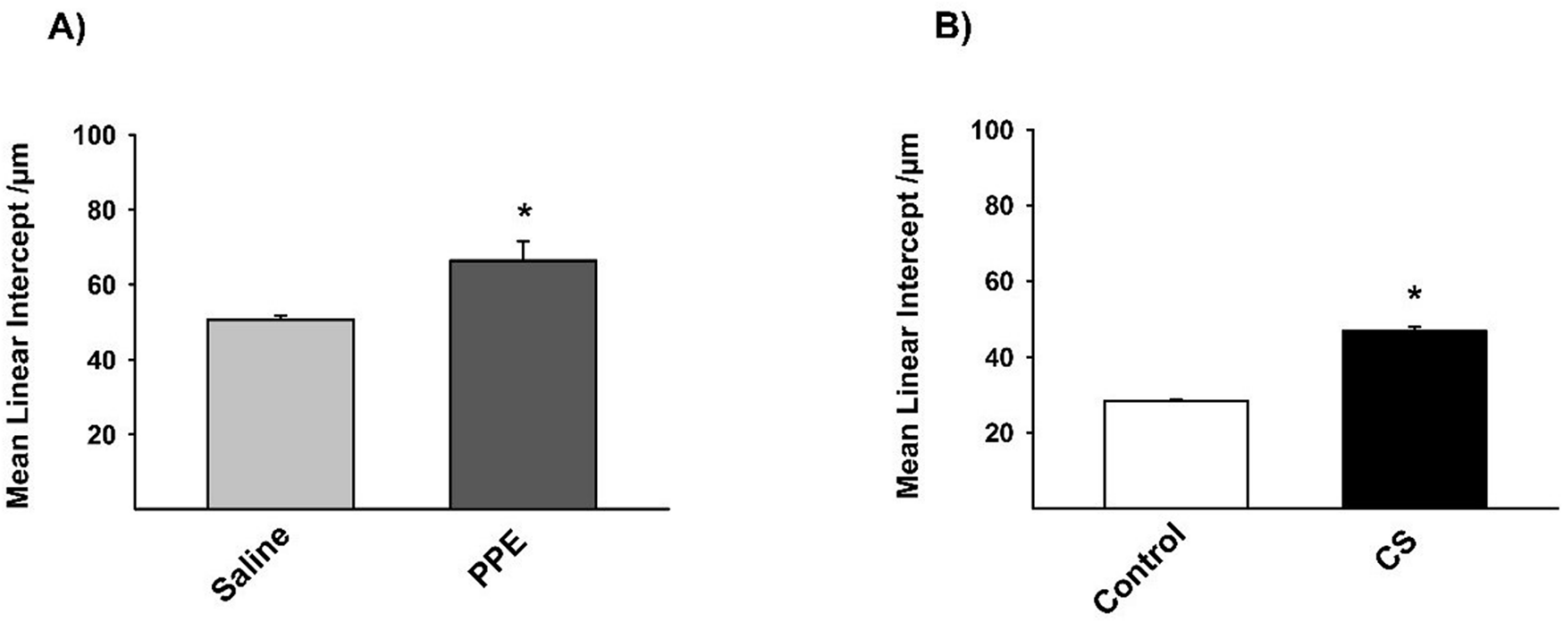
**Lm (A,B) values measured in experimental groups.** (A) *P=0.009, (B) *P=0.001. Results were expressed as the mean±s.e. Saline *n*=10; PPE *n*=8; control *n*=8; CS *n*=9.

**Fig. 2.**
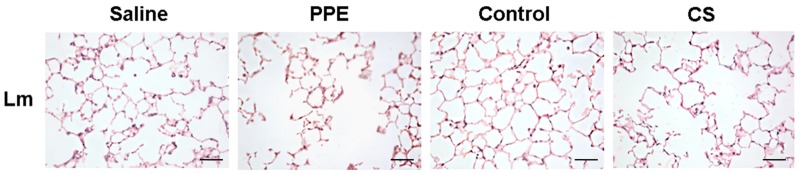
**Photomicrographs of Lm in the distal airspaces of saline, PPE, control and CS groups.** 200× magnification, scale bars: 100 μm.

The Lm revealed an increase in the two experimental models proposed, demonstrating the increase in the distal airspaces (Figs 1,2).

### Immunohistochemistry for total macrophages

**Fig. 3.**
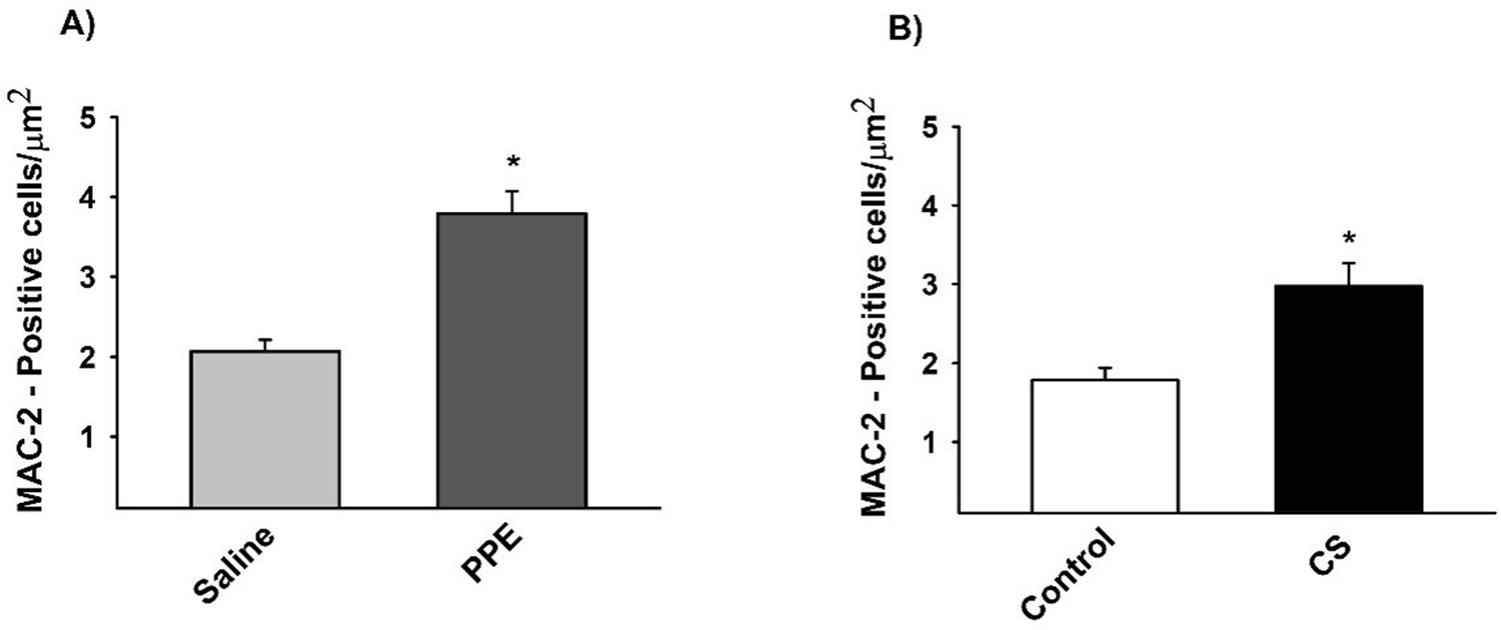
**The density of positive cells for MAC-2 (A,B).** (A) *P=0.001, (B) *P=0.001 compared to their respective controls. Values are expressed as the mean±s.e. Saline *n*=11; PPE *n*=10; control *n*=11; CS *n*=9.

**Fig. 4.**
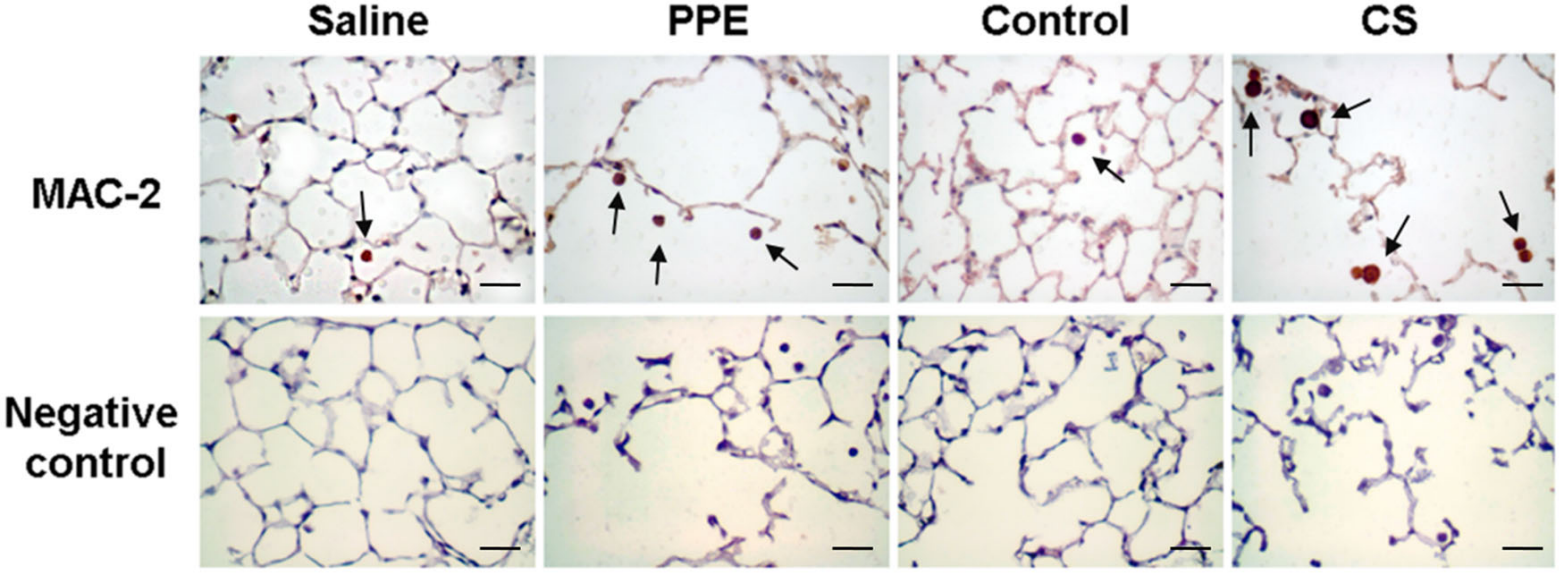
**Photomicrographs of positive cells for MAC-2 and their respective negative controls in lung parenchyma in saline, PPE, control and CS groups.** The arrows show the MAC-2+ cells in lung parenchyma. 400× magnification, scale bars: 50 μm.

The density of the MAC-2+ cells was increased in the PPE and CS groups compared to their respective controls (Figs 3,4).

### Immunohistochemistry for M1-like macrophages markers

**Fig. 5.**
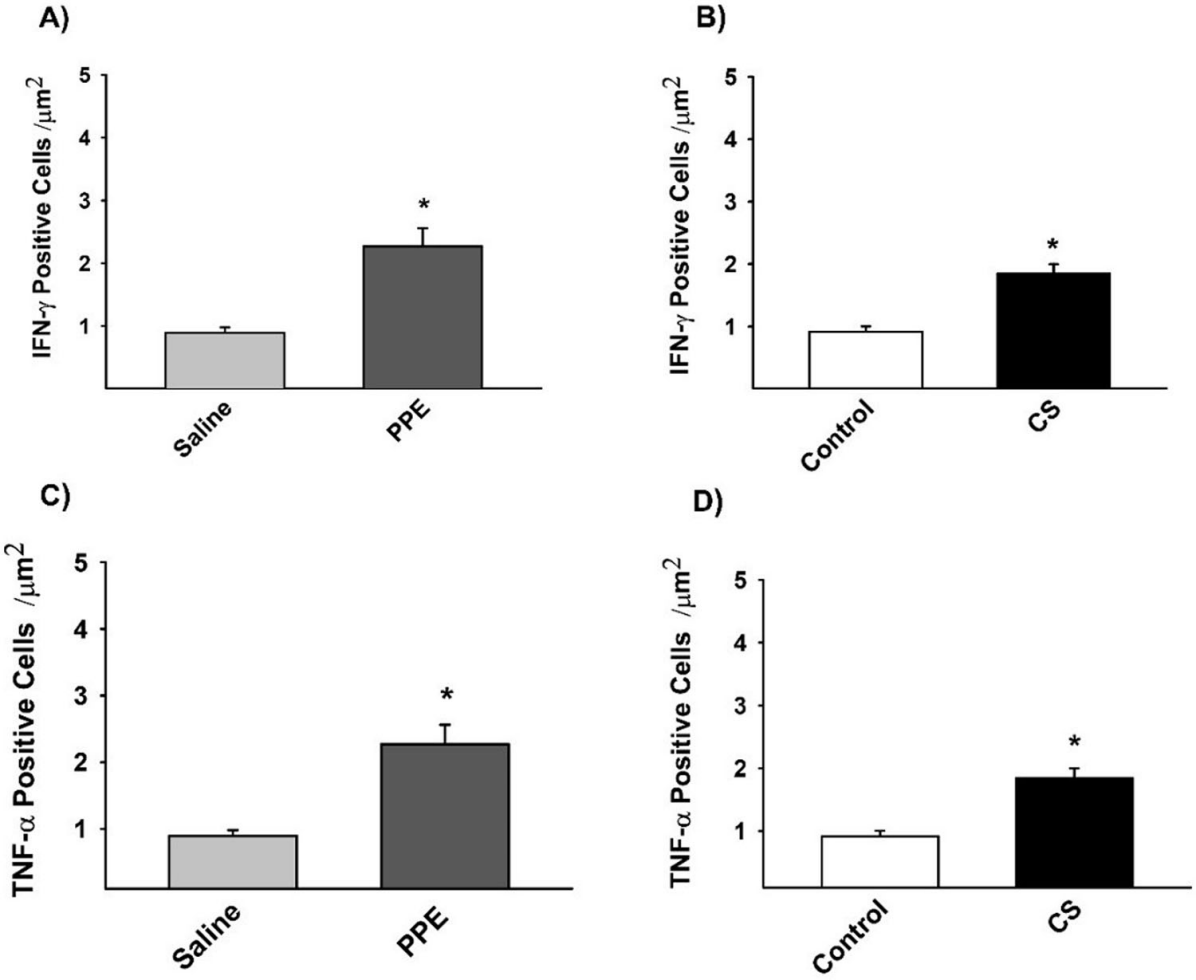
**The density of positive cells for IFN- γ (A,B) and TNF-α (C,D), M1-like macrophages markers.** (A) *P=0.004, (B) *P=0.001, (C) *P=0.037, (D) *P=0.001, compared to their respective controls. Values are expressed as the mean±s.e. IFN-γ: saline *n*=8; PPE *n*=10; control *n*=9; CS *n*=9. TNF-α: saline *n*=8; PPE *n*=10; control *n*=9; CS *n*=9.

The density of the positive cells involved in the differentiation of the M1-like macrophages demonstrated an increase in IFN-γ and TNF-α in the PPE and CS groups compared to their respective controls (Figs 5,7).

### Immunohistochemistry for M2-like macrophages markers

**Fig. 6.**
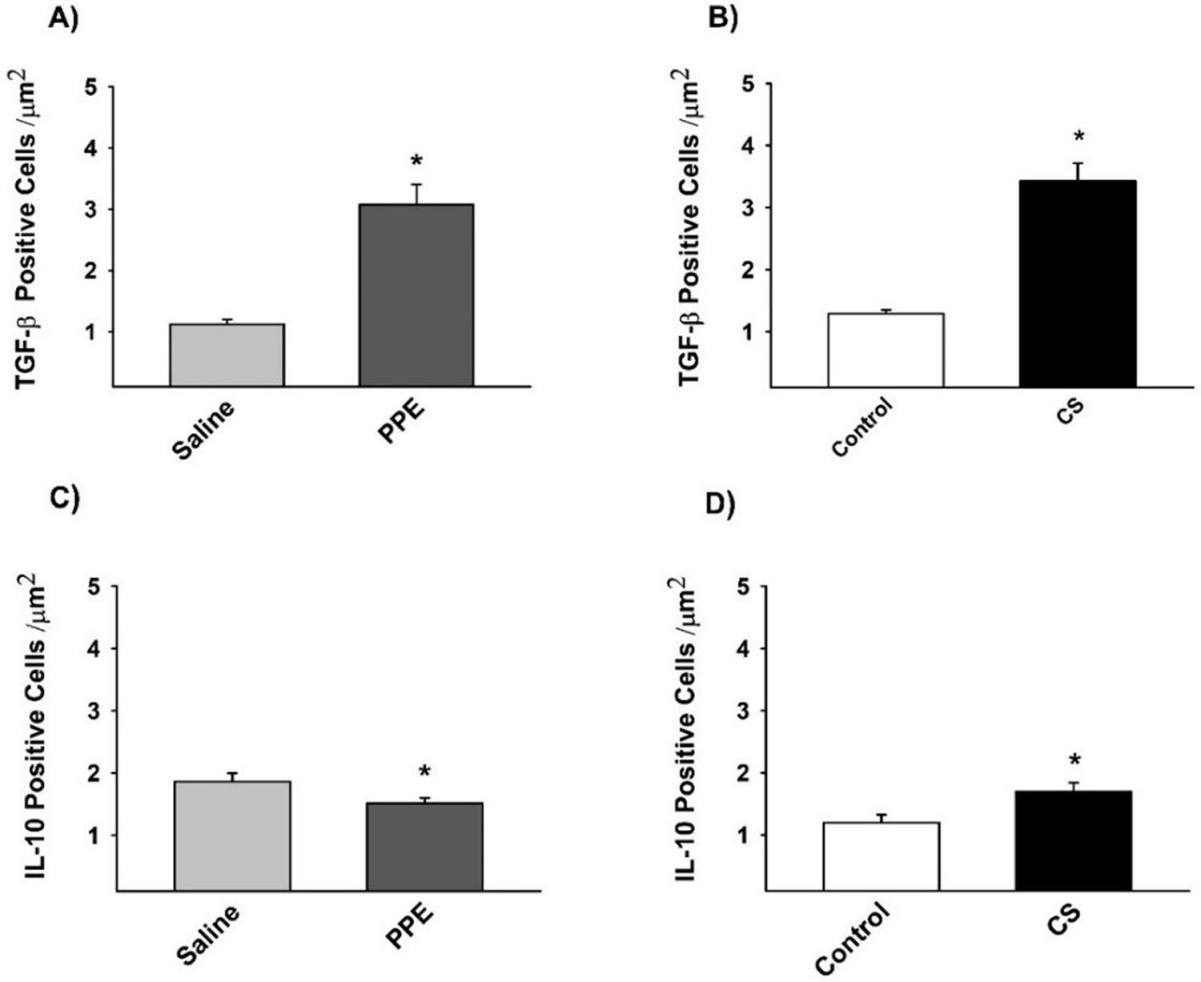
**The density of positive cells for TGF-β (A,B) and IL-10 (C,D), M2-like macrophages markers.** (A) *P=0.001, (B) *P=0.001, (C) *P=0.041, (D) *P=0.029, compared to their respective controls. Values are expressed as the mean±s.e. TGF-β: saline *n*=9; PPE *n*=8; control *n*=8; CS *n*=9. IL-10: saline *n*=8; PPE *n*=8; control *n*=7; CS *n*=7.

**Fig. 7.**
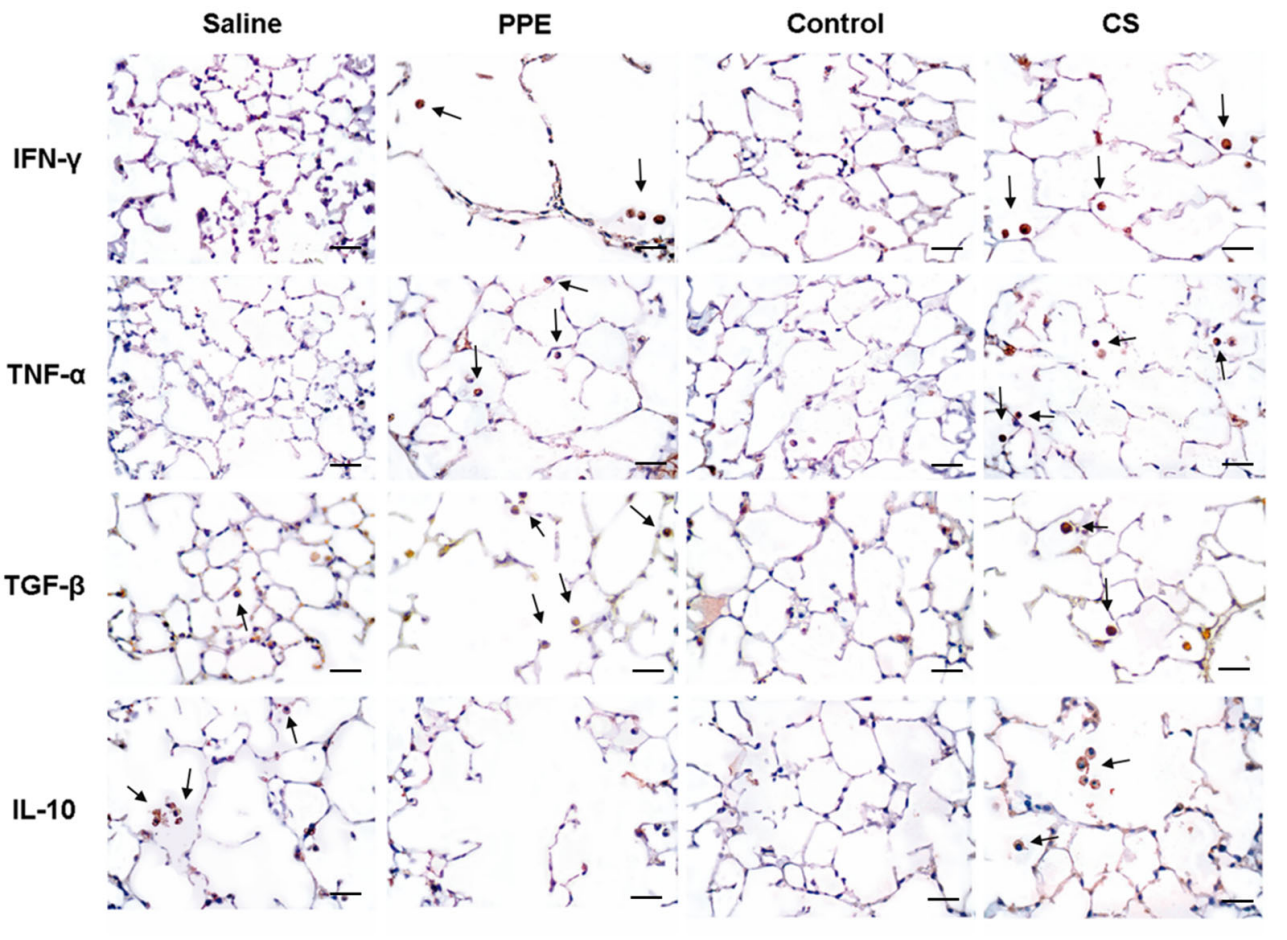
**Photomicrographs of positive cells for IFN-γ, TNF-α, TGF-β and IL-10 in lung parenchyma in saline, PPE, control and CS groups.** The arrows show IFN-γ+, TNF-α+, TGF-β+ and IL-10+ cells in lung parenchyma. 400× magnification, scale bars: 50 μm.

The density of TGF-β+ cells, a cytokine which characterizes M2-like macrophage, was increased in PPE and CS induced inflammation, while IL-10+ cells density was decreased in the PPE group compared to the saline group and increased in the CS group compared to the respective control (Figs 6,7).

### Cytokine analysis detected by ELISA

There was no expression of IL-12 and IFN-γ M1-like macrophage markers; however, we observed a decrease in the expression of IL-4 (Fig. 9A), IL-10 (Fig. 9C) and IL-13 (Fig. 9E), markers of M2-like macrophage, in the PPE group compared to the saline group. We found an increased expression of IL-10 (Fig. 9D) only in the CS group compared to the control group (Fig. 9).

**Fig. 8.**
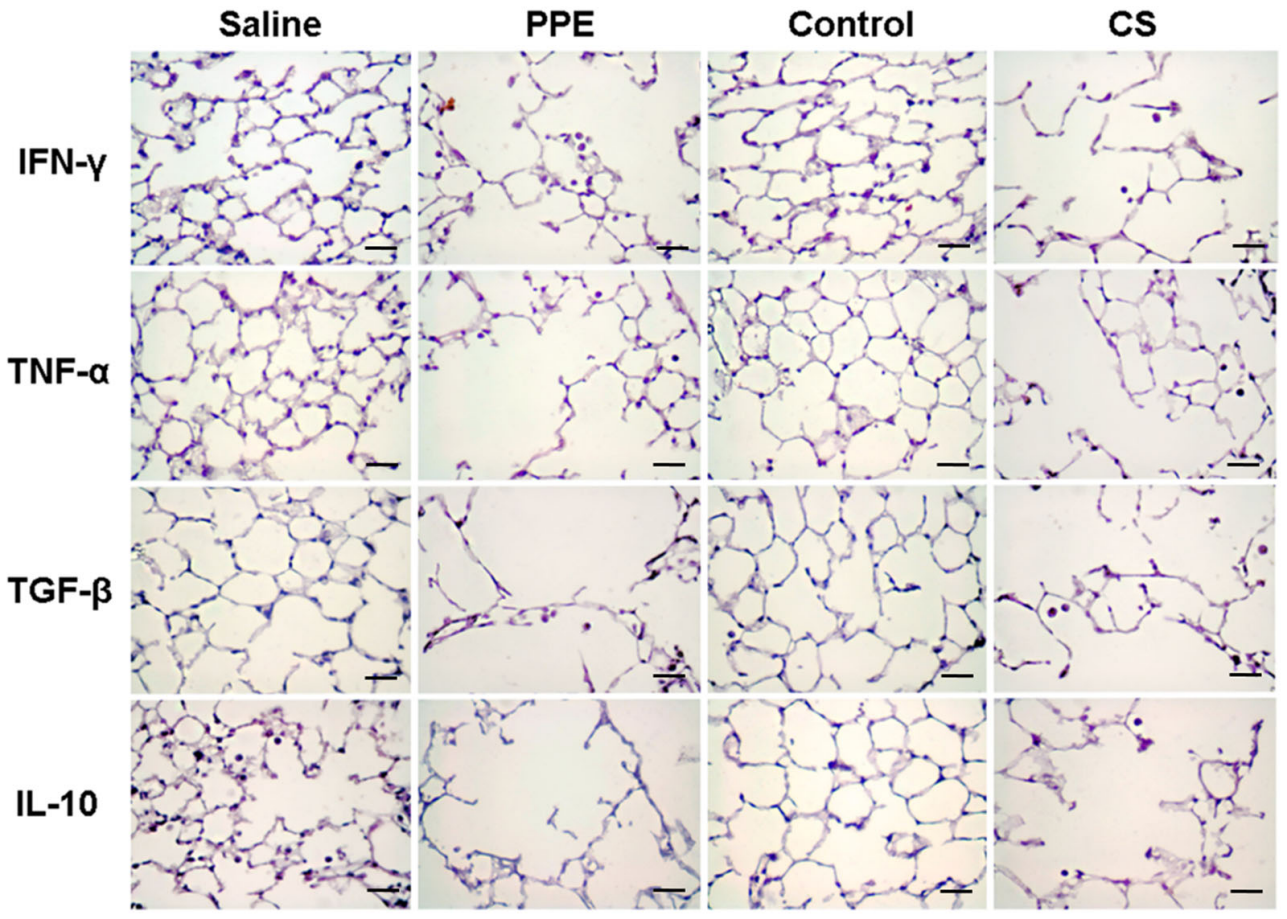
**Photomicrographs of negative control images for IFN-γ, TNF-α, TGF-β and IL-10 in lung parenchyma in saline, PPE, control and CS groups.** 400× magnification, scale bars: 50 μm.

**Fig. 9.**
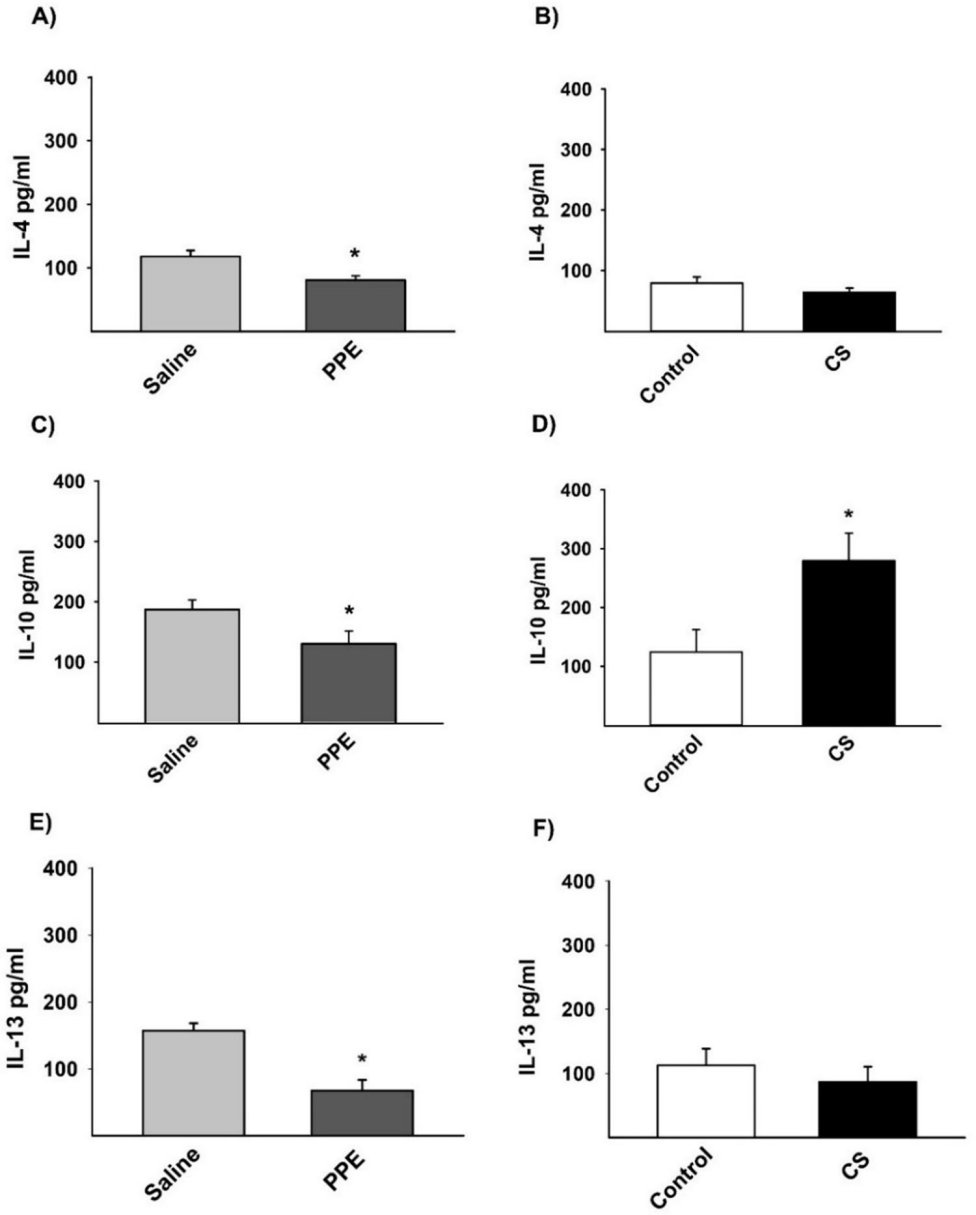
**Protein quantification of IL-4 (A,B), IL-10 (C,D), and IL-13 (E,F) by ELISA.** (A) *P=0.016, (C) *P=0.046, (D) *P=0.028, (E) *P=0.007, compared to their respective controls. Values are expressed as the mean±s.e. IL-4: saline *n*=10; PPE *n*=9; control *n*=8; CS *n*=6. IL-10: saline *n*=7; PPE *n*=8; control *n*=6; CS *n*=6. IL-13: saline *n*=6; PPE *n*=9; control *n*=6; CS *n*=7.

### RT-qPCR – markers of M1-like macrophages

**Fig. 10.**
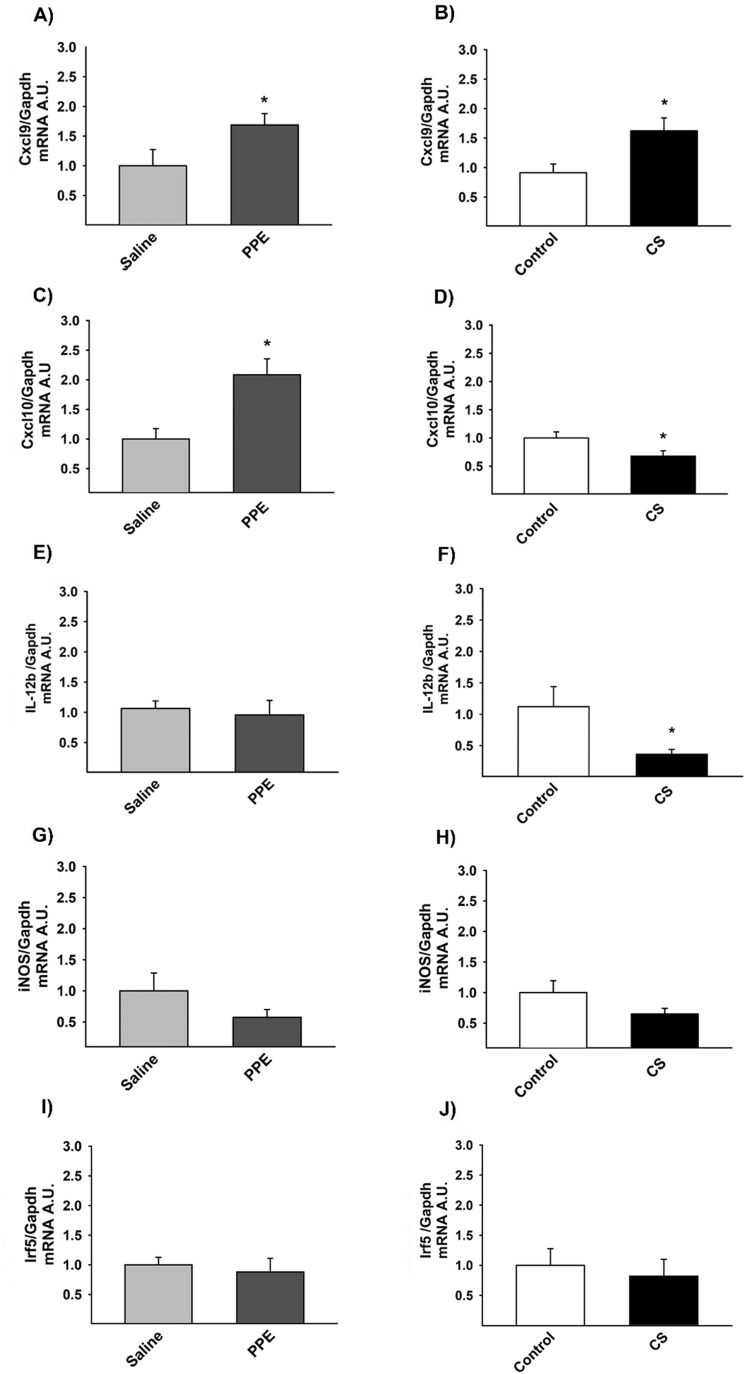
**Gene expression for *Cxcl9* (A,B), *Cxcl10* (C,D), Il-12b (E,F).** (A) *P=0.004, (B) *P=0.017, compared to their respective controls. (C) *P=0.001, (D) *P=0.036, compared to their respective controls. (F) *P=0.026, compared to the control group. We did not observe any significant results following *iNos* (G,H) and *Irf-5* (I,J) analysis. Values are expressed as the mean±s.e. CXCL9: saline *n*=14; PPE *n*=13; control *n*=11; CS *n*=13. CXCL10: saline *n*=14; PPE *n*=14; control *n*=11; CS *n*=16. IL-12b: saline *n*=13; PPE *n*=13; control *n*=10; CS *n*=11. iNOS: saline *n*=7; PPE *n*=4; control *n*=4; CS *n*=3. Irf5: saline *n*=14; PPE *n*=15; control *n*=12; CS *n*=17.

Gene expression in M1-like macrophages was expressed in arbitrary units. We found increased *Cxcl9* expression in the PPE group compared to respective control (Fig. 10A). However, *Cxcl10* gene expression was increased in both the PPE and CS groups compared to their respective controls (Fig. 10B). We observed decreased *Il-12b* gene expression in lungs from the CS group compared to the control group (Fig. 10E). There was no statistically significant difference in gene expression of *Irf5* (Fig. 10C) and *iNos* (Fig. 10D) between the different experimental groups (Fig. 10).

### RT-qPCR – markers of M2-like macrophages

**Fig. 11.**
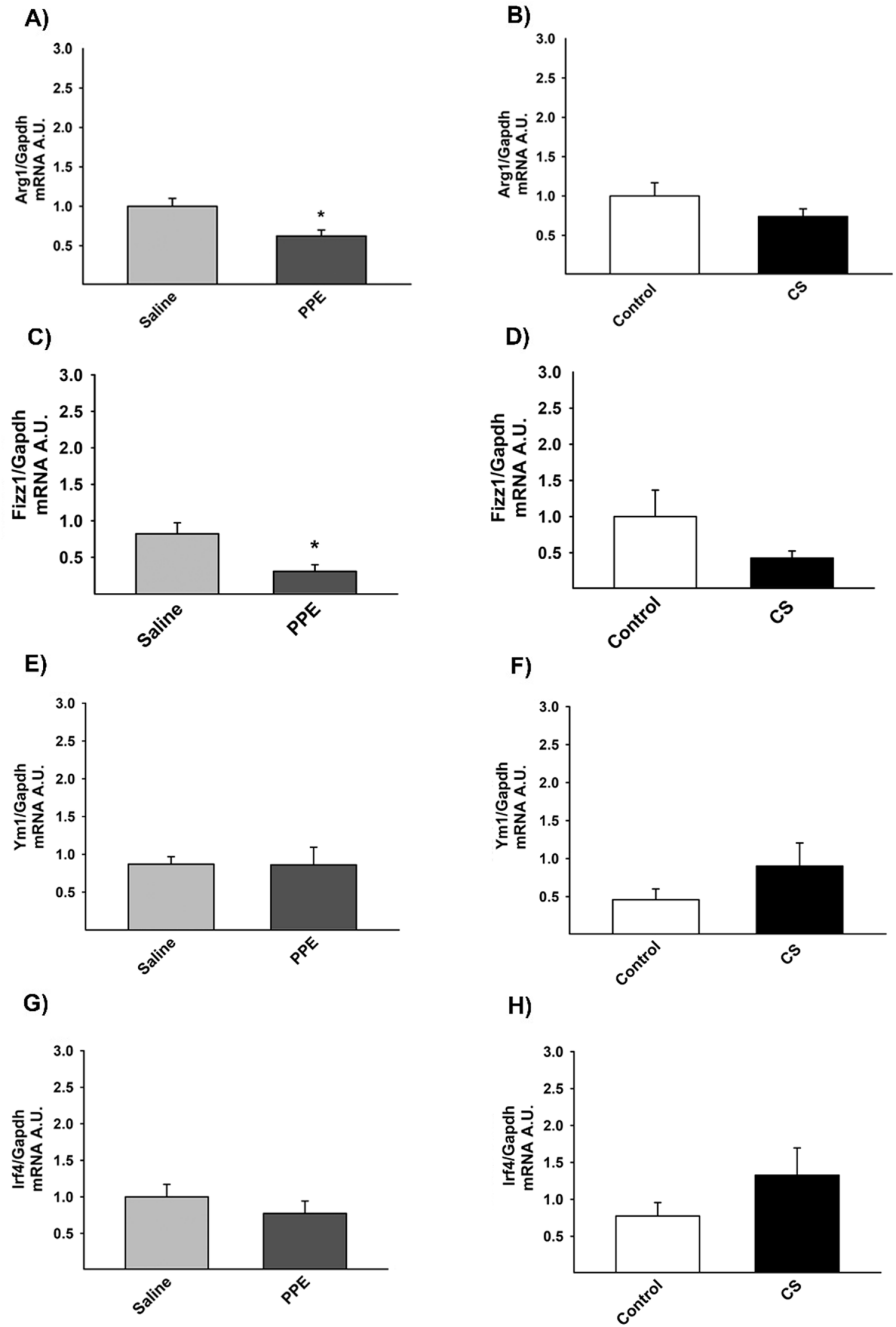
**Gene expression for *Arg1* (A,B), *Fizz1* (C,D).** (A)*P=0.005 compared to the saline group. (C) *P=0.001 compared to the saline group. No differences in *Ym1* and (E,F) *Irf4* (G,H) gene expression were detected. Values are expressed as the mean±s.e. Arg1: saline *n*=14; PPE *n*=15; control *n*=12; CS *n*=17. Fizz1: saline *n*=13; PPE *n*=14; control *n*=12; CS *n*=16. Ym1: saline *n*=13; PPE *n*=14; control *n*=9; CS *n*=16. Irf4: saline *n*=14; PPE *n*=14; control *n*=10; CS *n*=14.

The evaluation of gene expression for M2-like macrophages markers revealed a decrease for the expression of *Arg1* (Fig. 11A) and *Fizz1* (Fig. 11D) in the PPE group compared to the saline group. There was no significant difference between experimental groups for *Ym1* and *Irf4* gene expression (Fig. 11B,C).

## DISCUSSION

Our results showed differences in microenvironmental stimuli due to gene expression and due to the release of chemokine related to M1- and M2-like macrophage phenotypes in the CS and PPE-induced models. Whereas the CS-induced model showed M1 and M2-related genes and chemokines, the PPE-induced model showed a downregulation of M2- and an increase in M1-related genes and chemokines (Table 1).

**Table 1.**
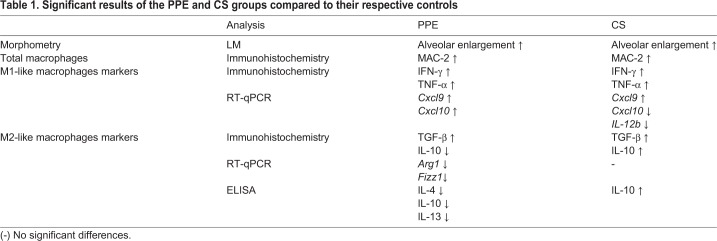
**Significant results of the PPE and CS groups compared to their respective controls**

Considering the structural changes in the lung parenchyma, we observed an alveolar enlargement in both models. However, a higher mean linear intercept was observed in the PPE model, reinforcing previous findings that describe greater alveolar destruction by proteases than that in the CS-induced models (Wright et al., 2008; Lopes et al., 2013). There were no significant differences, despite the age difference, when we compared the control group (saline, 28 day; control, 3 month) at the end of their respective exposures.

After 28 days of PPE instillation, we observed a decrease in the majority of M2-related genes and chemokines such as IL-4, IL-10, IL-13, *Arg*1, *Fizz1*. On the other hand, we found a higher density of TNF-α+, IFN-γ+ cells and increased gene expression of *Cxcl9* and *Cxcl10*, resulting in stimuli that induce M1-like macrophage polarizations.

Analysis of the interleukins in the CS model revealed that after 3 months of exposure, there was a decreased expression of *IL-12b*, IL-4, IL-13, *Fizz1* and *Arg1*, while we noted an increase in IL-10 expression, suggesting a polarization towards M2-like macrophage polarization.

Mantovani et al. (2013) described different subtypes of M2 phenotypes (M2a, M2b and M2c) with different functions. In the CS group, we observed an increase in IL-10 by immunohistochemistry and ELISA with a concomitant decrease in *IL-12b* gene expression associated with an increase in TNF-α+ cells in the parenchyma, suggesting the presence of an M2b polarized phenotype, which exert immunoregulatory functions and drive Th2 responses. Additionally, we detected an increase in TGF-β+ cells and IL-10 expression, which are characteristic of M2c-like macrophage and associated with immune response suppression and tissue remodeling.

In addition, CS exposure also induced a significant increase in chemokines and gene expression related to M1-like macrophage polarizations such as TNF-α, IFN-γ and *Cxcl9*.

Although the CS- and PPE-induced models are basically different, both models showed proteolysis and pulmonary inflammation and an increase in macrophages in the respiratory tract (Wright et al., 2008).

There is a large body of evidence attesting the importance of macrophages in the progression of the inflammatory response in COPD patients. Indeed, there is a positive correlation between COPD severity and the increase in macrophages in the airways (Vlahos and Bozinovski, 2014).

We showed, in both experimental models, an increase in the density of macrophages in the lung parenchyma and an increase in the inflammatory mediators that induce M1 polarization. However, M2-like related genes and chemokines were observed only in animals that were chronically exposed to CS.

Until now, there have been few studies describing macrophage polarization in COPD patients. Shaykhiev et al. (2009) showed that during the development of COPD, a suppression of host defenses and a decrease in M1 polarization occur, along with a concomitant increase in immunosuppressive factors such as IL-10 and TGF-β related to M2-like macrophage polarizations.

On the other hand, Hodge et al. (2011) described a mixed phenotype in the bronchoalveolar lavage fluid from COPD patients compared to healthy smokers. They also detected a defective activity to phagocyte apoptotic cells.

Macrophages display remarkable plasticity, changing their physiology in response to environmental stimuli (Hodge et al., 2011). During the development of COPD, both M1- and M2-like macrophage polarizations are present (Mosser and Edwards, 2008).

The increased M2 phenotype observed in COPD patients may reflect a deficiency in the control of the inflammatory process mediated by the phagocytosis of apoptotic cells. A deficiency in the removal of apoptotic cells improves the inflammatory process and the progression of this disease (Taraseviciene-Stewart and Voelkel, 2008).

Our results showed that the consequences of the CS-induced model were similar to those previously described in COPD patients, with both M1- and M2-related genes and chemokines being increased. This response is probably due to the chronic exposure to CS compared to the PPE-induced model that required a single elastase instillation.

It is important to note that, due to the anatomical and physiological differences between humans and other species, there is no ideal animal model that is able to mimic all features observed in human diseases (Shaykhiev et al., 2009; Taraseviciene-Stewart and Voelkel, 2008). Therefore, it is important to better characterize the physiological features of each animal model before choosing one for further investigations.

Considering our results, exposure to CS in rodents may be able to better reproduce some features of the innate immune response that have already been described in COPD patients compared to those in the PPE-induced model. Chronic exposure to CS resulted in an inflammatory process induced by an environmental stimulus related to the M1-like and M2-like polarized phenotypes.

## MATERIALS AND METHODS

### Animals

This study was approved by the Human and Animal Research Ethics Committee of the University of São Paulo School of Medicine under protocol number 136/2015 (São Paulo, Brazil). We used six- to eight-week-old male C57BL/6 mice (weighing 20–25 g) all animals received human care in compliance with the US National Institutes of Health Guide for the Care and Use of Laboratory Animals (NIH Publication no. 85–23, revised in 1996).

### Induction of emphysema

#### Porcine pancreatic elastase (PPE)-induced model

Animals were anesthetized with a combination of xylazine and ketamine (5 mg/kg and 40 mg/kg, respectively) to allow for intranasal instillation of 50 µl of type I PPE (E1250; Sigma-Aldrich, St. Louis, MO, USA) at a dose of 0.667 IU. The saline group received 50 µl of saline (0.9%) by intranasal instillation.

#### CS-induced model

We used a plastic box (28 l) with two inlets for filtered air and CS (Biselli et al., 2011). A flow meter was regulated using a Venturi system connected to a lit cigarette, allowing for suction of the CS into the box and maintaining the airflow at 2 l/min. A flow rate was set to produce carbon monoxide levels ranging from 250–350 ppm. We used approximately 12 (±1) commercially filtered cigarettes per day (Derby, Souza Cruz, Brazil; 0.8 mg of nicotine, 10 mg of tar and 10 mg of carbon monoxide per cigarette) with a total particulate matter concentration of 354.8±50.3 μg/m^3^/day. The exposures to CS were performed for 30 min/exposure, two times/day, 5 days/week for a period of 12 weeks. The control group were exposed to filtered air (Vlahos and Bozinovski, 2014).

### Experimental groups

The animals were divided into four different experimental groups: saline group; animals received intranasal instillation of saline (0.9%). PPE group; animals received intranasal instillation of PPE. Control group; animals were exposed to filtered air. CS group; animals were exposed to CS.

### Lung preparation

To remove the lungs, animals were euthanized under anesthesia (Thiopental 50 mg/kg). Lungs were infused with formalin through the trachea at a constant pressure of 20 cmH_2_O for 24 h and were embedded in paraffin and cut into 5 µm coronal sections for morphometry evaluation.

### Morphometry

For conventional morphometry, tissue samples were stained with H&E to perform the mean linear intercept (Lm) measurements. Lm was obtained by counting the number of times that the lines of the reticulum intercepted the alveolar walls using following equation: Lm=Ltotal/NI (Mosser and Edwards, 2008). We performed the Lm analysis in the distal areas of the parenchyma (peripheral airspaces).

### Immunohistochemistry

To perform immunohistochemistry, tissue sections were deparaffinized and hydrated. After blocking endogenous peroxidase activity, antigen retrieval was obtained under a high temperature using citrate buffer (pH=6.0). The primary antibodies used were: a rat monoclonal anti-MAC-2 (1:25.000, Cedarlane, Ontario, Canada), a rat monoclonal anti-IFN-γ (1:1000, Santa Cruz Biotechnology, USA), a rat monoclonal anti-TNF-α, (1:15,000, Santa Cruz Biotechnology), a goat polyclonal anti-IL-10 (1:50, Santa Cruz Biotechnology), a rabbit polyclonal anti-TGF-β (1:400, Santa Cruz Biotechnology). The following Vectastain ABC Kit (Vector Laboratories, Burlingame, USA) was used in conjunction with a species-specific secondary antibody (Nichirei Biosciences, Tokyo, Japan), and the sections were stained using the chromogen diaminobenzidine (Sigma-Aldrich). The sections were counterstained with Harris Hematoxylin (Merck, Darmstadt, Germany). For negative controls (Fig. 8) the primary antibody was omitted from the procedure (Vlahos and Bozinovski, 2014) and bovine serum albumin was used instead in tissue samples.

The density of MAC-2 +, IFN-γ+, TNF-α+, IL-10+, and TGF-β+ cells in lung parenchyma were quantified. For this, we used the same reticule of 100 points and 50 straight lines with a known area (62.500 μm^2^) under 400× magnification. The area of the alveolar tissue in each field was calculated according to the number of points that intercepted the alveolar septa and determined with the following equation: positive cell density=number of positive cells/number of points that were parenchymatic, the result was expressed as cells/μm^2^ (Margraf et al., 1991).

### Cytokine analysis

Levels of IFN-γ, IL-12 (cytokines characteristic of M1-like macrophages); IL-4; IL-10 and IL-13 (cytokines characteristic of M2-like macrophages) were determined by ELISA (OptEIA, BD Pharmingen, Oxford, UK) in bronchoalveolar lavage using microplates (Costar, Cambridge, MA, USA) for each cytokine sensitized with specific monoclonal antibodies. After washing and distribution of the samples, specific antibodies conjugated to biotin were added to the different cytokines. For development of the bond, a developer solution containing streptavidin-peroxidase, substrate and a chromogen enzyme conjugate were added. The reaction was read at 450 nm in a M2 spectrophotometer (Spectramax L, Molecules Devices, CA, USA). Sample concentrations were calculated using the standard curves obtained with the recombinant cytokines and the result was expressed per pg/ml.

### Real time PCR

RNA extraction, reverse transcription and quantitative real-time PCR (qRT-PCR) were performed in lung homogenate. Total RNA from 3T3-L1 cell lysates was extracted using TRIzol (Invitrogen Life Technologies, CA, USA), analyzed for quality on agarose gel and read using absorbance ratios of 260/280 nm and 260/230 nm, and reverse transcribed to cDNA using the SuperScript III cDNA kit (Invitrogen Life Technologies). Gene expression was evaluated by qRT-PCR using a Rotor Gene (Qiagen, Roermond, Netherlands) and SYBR Green as the fluorescent dye (Qiagen) with GAPDH as the housekeeping gene. The reaction conditions were as follows: 95°C for 5 min, then 40 cycles of 95°C for 5 s and 60°C for 10 s. PCR products were run on agarose gels to confirm the size of the fragment and specificity of amplification. the primers used were as follows: *Irf-5* (5′-3′ sense: AATACCCCACCACCTTTTGA; 5′-3′ antisense: TTGAGATCCGGGTTTGAGAT); *Irf-4* (5′-3′ sense: CAAAGCACAGAGTCACCTGG; 5′-3′ antisense: TGCAAGCTCTTTGACACACA); *Fizz-1* (5′-3′ sense: TCCCAGTGAATACTGATGAGA; 5′-3′ antisense: CCACTCTGGATCTCCCAAGA); *Ym-1* (5′-3′ sense: GGGCATACCTTTATCCTGAG; 5′-3′ antisense: CCACTGAAGTCATCCATGT); *Cxcl-9* (5′-3′ sense: TGCACGATGCTCCTGCA; 5′-3′ antisense: AGGTCTTTGAGGGATTTGTAGTGG); *Cxcl-10* (5′-3′ sense: GACGGTCCGCTGCAACTG; 5′-3′ antisense: GCTTCCCTATGGCCCTCATT); *Il-12b* (5′-3′ sense: CGCAAGAAAGAAAAGATGAAGGAG; 5′-3′ antisense: TTGCATTGGACTTCGGTAGATG); *Arg-1* (5′-3′ sense: GCACTCATGGAAGTACACGAGGAC; 5′-3′ antisense: CCAACCCAGTGATCTTGACTGA); *iNos* (5′-3′ sense: ACCCGTCCACAGTATGTGAGGA; 5′-3′ antisense: GACCCCAAGCAAGACTTGGACT) and *Gapdh* (5′-3′ sense: CCACCACCCTGTTGCTGTAG; 5′-3′ antisense: CTTGGGCTACACTGAGGACC).

### Statistical analysis

Statistical analysis was performed using the SigmaStat program (Systat Software, San Jose, CA, USA; version 11.0). A *t*-test or Mann–Whitney test was used depending on the normality of the data. The results are expressed as the means±s.e. and *P*-values <0.05 were considered as statistically significant.

## References

[BIO040808C1] Barnes P. J. (2004). Alveolar macrophages as orchestrators of COPD. *COPD* 1, 59-70. 10.1081/COPD-120028701 16997739

[BIO040808C2] Biselli P. J. C., Lopes F. D. T. Q. S., Moriya H. T., Rivero D. H. R. F., Toledo A. C., Saldiva P. H. N., Mauad T. and Martins M. A. (2011). Short-term exposure of mice to cigarette smoke and/or residual oil fly ash produces proximal airspace enlargements and airway epithelium remodeling. *Braz. J. Med. Biol. Res.* 44, 460-468. 10.1590/S0100-879X2011007500040 21445523

[BIO040808C3] Bonfield T. L. (2012). *Lung Diseases - Selected State of the Art Reviews* (ed. Irusen E. M.), Ch. 18, pp. 407-428. In Vivo Models of Lung Disease.

[BIO040808C4] Finkelstein R., Fraser R. S., Ghezzo H. and Cosio M. G. (1995). Alveolar inflammation and its relation to emphysema in smokers. *Am. J. Respir. Crit. Care. Med.* 152, 1666-1672. 10.1164/ajrccm.152.5.7582312 7582312

[BIO040808C5] Global Initiative for Chronic Obstructive Lung Disease 2019 (GOLD 2019)—Global Strategy for the Diagnosis, Management and Prevention of COPD [Internet] (2019). Available from: https://goldcopd.org/wp-content/uploads/2018/11/GOLD-2019-v1.7-FINAL-14Nov2018-WMS.pdf.

[BIO040808C6] Goerdt S. and Orfanos C. E. (1999). Other functions, other genes: alternative activation of antigen-presenting cells. *Immunity* 10, 137-142. 10.1016/S1074-7613(00)80014-X 10072066

[BIO040808C7] Grashoff W. F., Sont J. K., Sterk P. J., Hiemstra P. S., de Boer W. I., Stolk J., Han J. and van Krieken J. M. (1997). Chronic obstructive pulmonary disease: role of bronchiolar mast cells and macrophages. *Am. J. Pathol.* 151, 1785-1790. 9403729 PMC1858350

[BIO040808C8] Hodge S., Hodge G., Ahern J., Jersmann H., Holmes M. and Reynolds P. N. (2007). Smoking alters alveolar macrophage recognition and phagocytic ability: implications in chronic obstructive pulmonary disease. *Am. J. Respir. Cell Mol. Biol.* 37, 748-755. 10.1165/rcmb.2007-0025OC 17630319

[BIO040808C9] Hodge S., Matthews G., Mukaro V., Ahern J., Shivam A., Hodge G., Holmes M., Jersmann H. and Reynolds P. N. (2011). Cigarette smoke-induced changes to alveolar macrophage phenotype and function are improved by treatment with procysteine. *Am. J. Respir. Cell Mol. Biol.* 44, 673-681. 10.1165/rcmb.2009-0459OC 20595463

[BIO040808C10] Hogg J. C., Chu F., Utokaparch S., Woods R., Elliott W. M., Buzatu L., Cherniack R. M., Rogers R. M., Sciurba F. C., Coxson H. O. et al. (2004). The nature of small-airway obstruction in chronic obstructive pulmonary disease. *N. Engl. J. Med.* 350, 2645-2653. 10.1056/NEJMoa032158 15215480

[BIO040808C11] Lopes F. D., Toledo A. C., Olivo C. R., Prado C. M., Leick E. A., Medeiros M. C., Santos A. B., Garippo A., Martins M. A. and Mauad T. (2013). A comparative study of extracellular matrix remodeling in two murine models of emphysema. *Histol. Histopathol.* 28, 269-276. 10.14670/HH-28.269 23275309

[BIO040808C12] Mahadeva R. and Shapiro S. D. (2002). Chronic obstructive pulmonary disease * 3: experimental animal models of pulmonary emphysema. *Thorax* 57, 908-914. 10.1136/thorax.57.10.908 12324680 PMC1746206

[BIO040808C13] Mantovani A., Sica A., Sozzani S., Allavena P., Vecchi A. and Locati M. (2004). The chemokine system in diverse forms of macrophage activation and polarization. *Trends Immunol.* 25, 677-686. 10.1016/j.it.2004.09.015 15530839

[BIO040808C14] Mantovani A., Biswas S. K., Galdiero M. R., Sica A. and Locati M. (2013). Macrophage plasticity and polarization in tissue repair and remodelling. *J. Pathol.* 229, 176-185. 10.1002/path.4133 23096265

[BIO040808C15] Margraf R., Tomashefski J. F. Jr., Bruce M. C. and Dahms B. B. (1991). Morphometric analysis of the lung in bronchopulmonary dysplasia. *Am. Rev. Respir. Dis.* 143, 391-400. 10.1164/ajrccm/143.2.391 1990959

[BIO040808C16] Mosser D. M. and Edwards J. P. (2008). Exploring the full spectrum of macrophage activation. *Nat. Rev. Immunol.* 8, 958-969. 10.1038/nri2448 19029990 PMC2724991

[BIO040808C17] Shaykhiev R., Krause A., Salit J., Strulovici-Barel Y., Harvey B.-G., O'Connor T. P. and Crystal R. G. (2009). Smoking-dependent reprogramming of alveolar macrophage polarization: implication for pathogenesis of chronic obstructive pulmonary disease. *J. Immunol.* 183, 2867-2883. 10.4049/jimmunol.0900473 19635926 PMC2873685

[BIO040808C18] Taraseviciene-Stewart L. and Voelkel N. F. (2008). Molecular pathogenesis of emphysema. *J. Clin. Invest.* 118, 394-402. 10.1172/JCI31811 18246188 PMC2214683

[BIO040808C19] Vlahos R. and Bozinovski S. (2014). Role of alveolar macrophages in chronic obstructive pulmonary disease. *Front. Immunol.* 5, 435 10.3389/fimmu.2014.00435 25309536 PMC4160089

[BIO040808C20] Vlahos R., Bozinovski S., Gualano R. C., Ernst M. and Anderson G. P. (2006). Modelling COPD in mice. *Pulm. Pharmacol. Ther.* 19, 12-17. 10.1016/j.pupt.2005.02.006 16286233

[BIO040808C21] Wright J. L., Cosio M. and Churg A. (2008). Animal models of chronic obstructive pulmonary disease. *Am. J. Physiol. Lung Cell. Mol. Physiol.* 295, L1-L15. 10.1152/ajplung.90200.2008 18456796 PMC2494776

